# Optically Programmable Smart WSe_2_/hBN Heterostructure Gas Sensors

**DOI:** 10.1021/acsami.5c09390

**Published:** 2025-08-12

**Authors:** Ayaz Ali, Prashant Bisht, Matthias Schrade, Wen Xing, Per Erik Vullum, Takashi Taniguchi, Kenji Watanabe, Bodh Raj Mehta, Branson D. Belle

**Affiliations:** † Department of Cybernetics, Nanotechnology and Data Processing, Faculty of Automation Control, Electronics and Computer Science, Silesian University of Technology, Akademicka 16, 44-100 Gliwice, Poland; ‡ Department of Smart Sensor Systems, SINTEF DIGITAL, Forskningsveien 1, Oslo 0373, Norway; § School of Advanced Materials Science and Engineering, Sungkyunkwan University, Suwon 16419, Republic of Korea; ∥ Department of Sustainable Energy Technology, 6298 SINTEF, Forskningsveien 1, Oslo 0373, Norway; ⊥ Department of Materials and Nanotechnology, SINTEF, Høgskoleringen 5, Trondheim 7034, Norway; # Research Center for Materials Nanoarchitectonics, 52747 National Institute for Materials Science, 1-1 Namiki, Tsukuba 305-0044, Japan; ∇ Research Center for Electronic and Optical Materials, National Institute for Materials Science, 1-1 Namiki, Tsukuba 305-0044, Japan; ○ Department of Physics, 28817 Indian Institute of Technology Delhi, New Delhi 110016, India; ◆ Directorate of Research, Innovation and Development, Jaypee Institute of Information Technology, Noida, U.P. 201309, India; ¶ Centre for Oceanography and the Blue Economy, 62707 University of the West Indies, Five Islands, Antigua and Barbuda

**Keywords:** two-dimensional materials, heterostructures, field-effect transistor (FET), gas sensor, kelvin probe force microscopy (KPFM)

## Abstract

Highly sensitive and energy-efficient gas sensors are essential for real-time environmental monitoring and air quality assessment. In this work, we present an optically programmable gas sensor based on WSe_2_/hBN heterostructure transistors for NO_*x*_ detection. The hBN interfacial layer enhances device performance by reducing charge trapping and improving transport, enabling the WSe_2_/hBN configuration to achieve a higher sensing response and faster recovery than WSe_2_/SiO_2_ devices. To understand the sensing mechanism, in situ Kelvin probe force microscopy (KPFM) was used, revealing that NO_*x*_ adsorption at the metal/semiconductor interface modulates the Schottky barrier height (SBH), which governs charge transport and gas sensitivity. Furthermore, we demonstrate that UV-induced charge modulation allows dynamic control of the sensor response, offering a tunable and reversible method for optimizing gas detection. This study highlights the potential of heterostructure engineering and optoelectronic modulation in developing next-generation, low-power, smart gas sensors for environmental monitoring applications.

## Graphical abstract

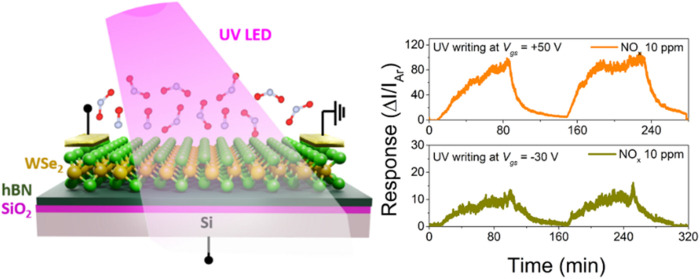

## 1 Introduction

Environmental monitoring technologies have garnered significant attention in recent years due to the alarming rise in global air pollution levels.^1,2^ Exposure to hazardous air pollutants, such as nitrogen oxides (NO_*x*_) and carbon monoxide (CO), poses severe risks to human health and has detrimental effects on the global environment, causing over 4.2 million deaths annually.^3^ Air pollution exacerbates conditions for individuals suffering from asthma and other respiratory disorders, highlighting the urgent need for effective environmental monitoring to safeguard public health and the ecosystem.

Current technologies for monitoring air pollution include sensors based on optical spectroscopy, which monitor the absorption of specific wavelengths in the UV–vis-NIR range, caused by electronic excitations or molecular vibrations in the analyte gas and solid-state devices.^4^ Optical gas sensors analyze the signals using photoacoustic spectroscopy or Fourier-transform infrared spectroscopy (FTIR).^4^ However, despite their excellent sensitivity, selectivity and fast response time, spectroscopic methods are hampered by their high cost, bulky and complex instrumentation making them impractical for widespread deployment.^4–6^ Solid-state gas sensors like chemoresistive or field-effect transistor (FET) sensors are versatile in terms of sensing mechanism, require simple instrumentation, and can be easily miniaturized for next-generation IoT application.^7–9^ In the last few decades, chemoresistive gas sensors based on metal oxide MOS (such as MoO_3_, WO_3_, TiO_2_, and SnO_2_) nanostructures have drawn immense attention.^10–13^ However, they typically require high operating temperatures (200–400 °C) to activate oxygen ad-ions, leading to high energy consumption.^14,15^

2D TMDCs based chemoresistive gas sensors have demonstrated high sensing response and limit of detection at room temperature which may be attributed to their atomic thickness, which provides a high specific surface area for enhanced adsorption and excellent electronic properties that enable superior transduction capabilities.^16^ Additionally, their peculiar mechanical properties enable integration into a flexible sensing system, making them ideal for next-generation application. In addition to TMDC-based devices, other emerging 2D materials have shown promise for chemoresistive gas sensing. Notably, borophene, an atomically thin allotrope of boron, has demonstrated exceptional sensitivity toward NO_2_ due to its high surface activity. Recent studies have reported that borophene-based sensors exhibit fast response/recovery times and low detection limits for NO_2_, highlighting their potential in environmental monitoring applications.^17–19^

Recently, 2D TMDC-based FETs have demonstrated immense potential in sensing trace amounts of gas and volatile organic compounds (VOC) at room temperature with a limit of detection up to a few ppb.^20^ FET gas sensors detect analyte gases by inducing changes in their conductance, threshold voltage, subthreshold swing, *I*_on_/*I*_off_ ratio, etc. Hence, by feeding multiple analysis parameters of the same exposure event into a Machine Learning (ML) algorithm, the selectivity and sensitivity of these FET sensors can be greatly increased.^21–24^ Notwithstanding, understanding the fundamental gas-matter interaction and the corresponding gas sensing mechanism is an important task to enhance the properties of the 2D TMD gas sensor.

Currently, most studies correlate FET sensing behavior with material properties like morphology, defects, functionalization, etc. to hypothesize the underlying sensing mechanism. However, operando and in situ gas sensing techniques can significantly enhance the understanding of sensing mechanisms by monitoring real-time changes in the physical properties of 2D TMDC and its impact on the device properties.^25^ For example, Cho et al. used in situ photoluminescence spectroscopy, revealing a direct charge transfer mechanism between the MoS_2_ film and gas molecules of NH_3_ and NO_2_.^26^ Similarly, Jensen et al. employed operando near ambient pressure XPS (NAP-XPS) on a monolayer MoS_2_ FET to investigate its charge transfer-based NO_*x*_ sensing.^27^ Recently, Bisht et al. employed in situ Kelvin Probe Force Microscopy (KPFM) to demonstrate the real-time changes in the work function of SnS thin films upon exposure to NO_*x*_ gas.^28^ Moreover, this study also substantiated the enhanced NO_2_ response and selective H_2_ sensing in Ag and Pd nanoparticle decorated SnS thin films, respectively, confirming the catalytic and doping effects of these nanoparticles. While nanoparticle-based doping has been effective in enhancing gas sensing performance, it also introduces limitations such as stability issues, surface contamination, and restricted tunability. Additionally, charge transfer dopants often induce carrier scattering, degrading mobility and overall device performance. In contrast, photoinduced doping presents a reversible and noninvasive alternative, offering dynamic control over charge carrier concentration while preserving intrinsic electronic properties.

In this work, we introduce an optically tunable gas sensing platform based on WSe_2_/hBN heterostructure FETs for NO_*x*_ detection. Compared to conventional WSe_2_/SiO_2_ devices, the WSe_2_/hBN configuration exhibits significantly enhanced sensing performance, including higher sensitivity, and faster response. To gain deeper insights into the sensing mechanism, we performed in situ Kelvin probe force microscopy (KPFM) imaging in the presence of NO_*x*_, providing direct experimental evidence that gas adsorption at the contact interface modulates the Schottky barrier, influencing charge transport dynamics. Furthermore, we demonstrate that the WSe_2_/hBN heterostructure enables photoinduced doping under UV illumination, offering a reversible and tunable method for optimizing gas detection.

## 2 Results and Discussions

### 2.1 Device Structure and Electrical Performance Comparison

Figure 1a illustrates the device structure of the WSe_2_ FETs utilized in this study. The WSe_2_ flake was transferred onto both a SiO_2_ substrate and an hBN flake using a dry transfer process (Figure S1),^29^ with the flake strategically positioned to span both regions equally. This design enables a direct comparison of the transport behavior between the WSe_2_/SiO_2_ and WSe_2_/hBN FET devices. To ensure consistency, a single WSe_2_ flake was used in the fabrication of both types of devices. Figure 1b presents an optical microscopy image of the devices. High-resolution transmission electron microscopy (HRTEM) images presented in Figure 1c and Supplementary Figure S2 confirm the number of WSe_2_ layers and the high-quality interfaces between WSe_2_ and hBN. The WSe_2_ flake used in this study has a thickness of approximately 6 nm, corresponding to ∼8 layers. Electrical measurements were conducted under dark conditions, and the transfer characteristics of the WSe_2_/SiO_2_ and WSe_2_/hBN FETs are shown in Figure 1d,1e, respectively. For effective comparison, the devices were designed with an identical channel length of 3.5 μm and width of 5 μm. The gate-source voltage (*V*_gs_) was varied from +50 to −50 V, and the drain-source current (*I*_ds_) was measured under a fixed drain-source voltage of 1 V. Both FETs exhibit unipolar p-type conduction. The WSe_2_/SiO_2_ device achieves a peak p-I_ON_ current of 0.56 μA at *V*_gs_ = −50 V and *V*_ds_ = 1 V, with a hole mobility of 2.94 cm^2^ V^–1^ s^–1^. In comparison, the WSe_2_/hBN device reaches a significantly higher p-I_ON_ current of 1.9 μA under the same gate and drain voltages, with an enhanced hole mobility of 11.83 cm^2^ V^–1^ s^–1^. Figure S3 shows the corresponding output curves, where both WSe_2_/SiO_2_ and WSe_2_/hBN devices show nonohmic behavior. Furthermore, the WSe_2_/hBN device exhibits significantly higher drain current (*I*_ds_ = 1.3 μA) compared to that of the WSe_2_/SiO_2_ counterpart (*I*_ds_ = 0.5 μA) under the same gate bias (*V*_gs_ = −50 V). The enhanced performance of the WSe_2_/hBN FET can be attributed to the presence of the hBN dielectric layer, which offers an atomically flat, defect-free interface, effectively minimizing charge trapping and surface roughness scattering. This improved interface reduces the density of interface states at the metal/semiconductor contact, thereby weakening Fermi level pinning and enabling more effective modulation of the Schottky barrier by the gate voltage. As a result, the WSe_2_/hBN device demonstrates a lower contact resistance and enhanced carrier injection.

**Figure 1:**
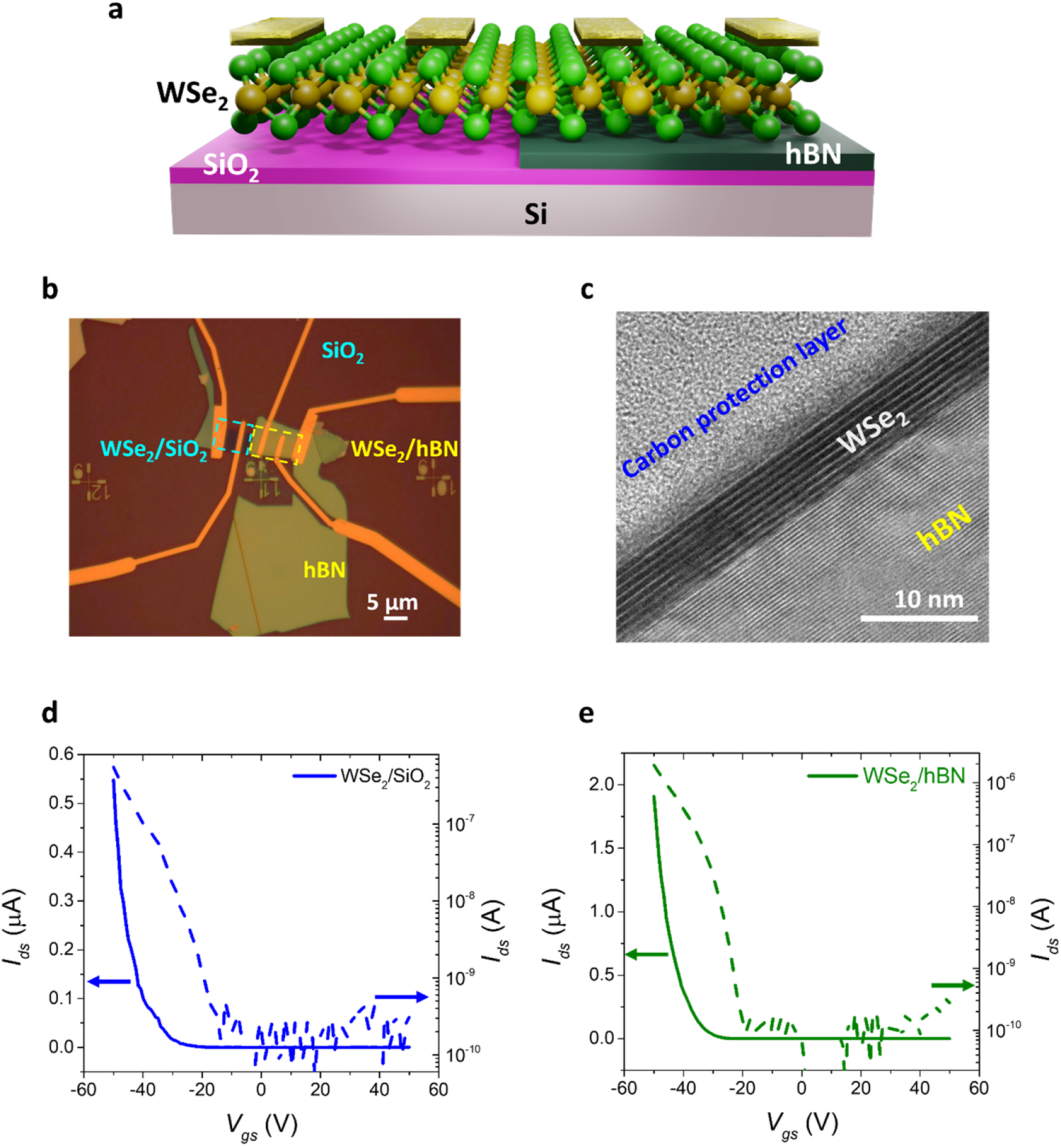
Electrical characterization of WSe_2_ field-effect transistors (FETs) fabricated on SiO_2_ and hBN substrates. (a) Schematic illustration of WSe_2_ FETs on SiO_2_ and hBN substrates. (b) Optical image of the fabricated devices. (c) High-resolution transmission electron microscopy (HRTEM) image of WSe_2_/hBN heterostructures fabricated using a dry transfer technique. (d, e) Transfer characteristics of WSe_2_/SiO_2_ and WSe_2_/hBN FETs at a drain-source voltage (*V*_ds_) of 1 V.

### 2.2 NO_*x*_ Sensing Performance of WSe_2_ FETs and Impact of hBN Interface Engineering

The electrical response of WSe_2_-based field-effect transistors (FETs) to NO_*x*_ exposure was investigated for both configurations (WSe_2_/SiO_2_ and WSe_2_/hBN) at room temperature. The incorporation of the hBN interfacial layer was found to play a crucial role in enhancing the sensing performance. The transfer characteristics of the WSe_2_/hBN FET exhibited a significant *V*_th_ shift from −25 to −12 V after exposure to 10 ppm of NO_*x*_, indicating a strong interaction between NO_*x*_ molecules and the WSe_2_ channel (Figure 2b). A logarithmic-scale plot is provided in the Supporting Information (Figure S4) for clearer visualization of the *V*_th_ shift. In contrast, the WSe_2_/SiO_2_ device exhibited a weaker response, likely due to the higher defect density and charge trapping at the WSe_2_/SiO_2_ interface (Figure S5). Furthermore, the cyclic response to varying NO_*x*_ concentrations (2 ppm to 10 ppm) revealed that the WSe_2_/hBN device exhibited a more pronounced and consistent response (Figure 2c and 2e), with a recovery time approximately two times faster (Figure S6). Additionally, both devices showed complete recovery after each sensing cycle, indicating efficient gas desorption at room temperature, a highly desirable characteristic. The sensing mechanism of NO_*x*_ in WSe_2_-based FETs is attributed to the strong electron-withdrawing nature of NO_*x*_ molecules, which act as p-type dopants upon adsorption on the WSe_2_ surface. When NO_*x*_ molecules physisorb or chemisorb onto the semiconductor channel, they extract electrons from WSe_2_, leading to a shift in the Fermi level and an increase in the hole concentration. This charge transfer process enhances the p-type conductivity of the WSe_2_ channel, which manifests as an increase in the drain current (Figure 2b). Additionally, the presence of NO_*x*_ at the contact interfaces modulates the Schottky barrier, enhancing the hole injection by modifying the metal work function. The effect of NO_*x*_ adsorption and desorption is more pronounced in the WSe_2_/hBN device. This enhancement in the sensing response can be directly attributed to the suppression of interfacial trap states and reduction in Coulombic scattering at the WSe_2_/hBN interface, which stabilizes the carrier transport and facilitates efficient charge transfer during NO_*x*_ gas exposure and recovery, thereby improving both kinetic response and signal linearity. This is further evidenced by the much faster recovery time in the WSe_2_/hBN device, while the WSe_2_/SiO_2_ device shows a significantly slower recovery (Figure S6). The extended recovery time observed in the SiO_2_-supported device is attributed to interfacial charge trapping and defect-mediated adsorption sites, which hinder the complete desorption of NO_*x*_ molecules in a short time. The observed trends in the NO_*x*_ response as a function of concentration confirm the enhanced sensitivity of the hBN-supported device (Figure 2d and 2f), highlighting the importance of dielectric engineering in optimizing the gas sensing characteristics of 2D material-based transistors.

**Figure 2:**
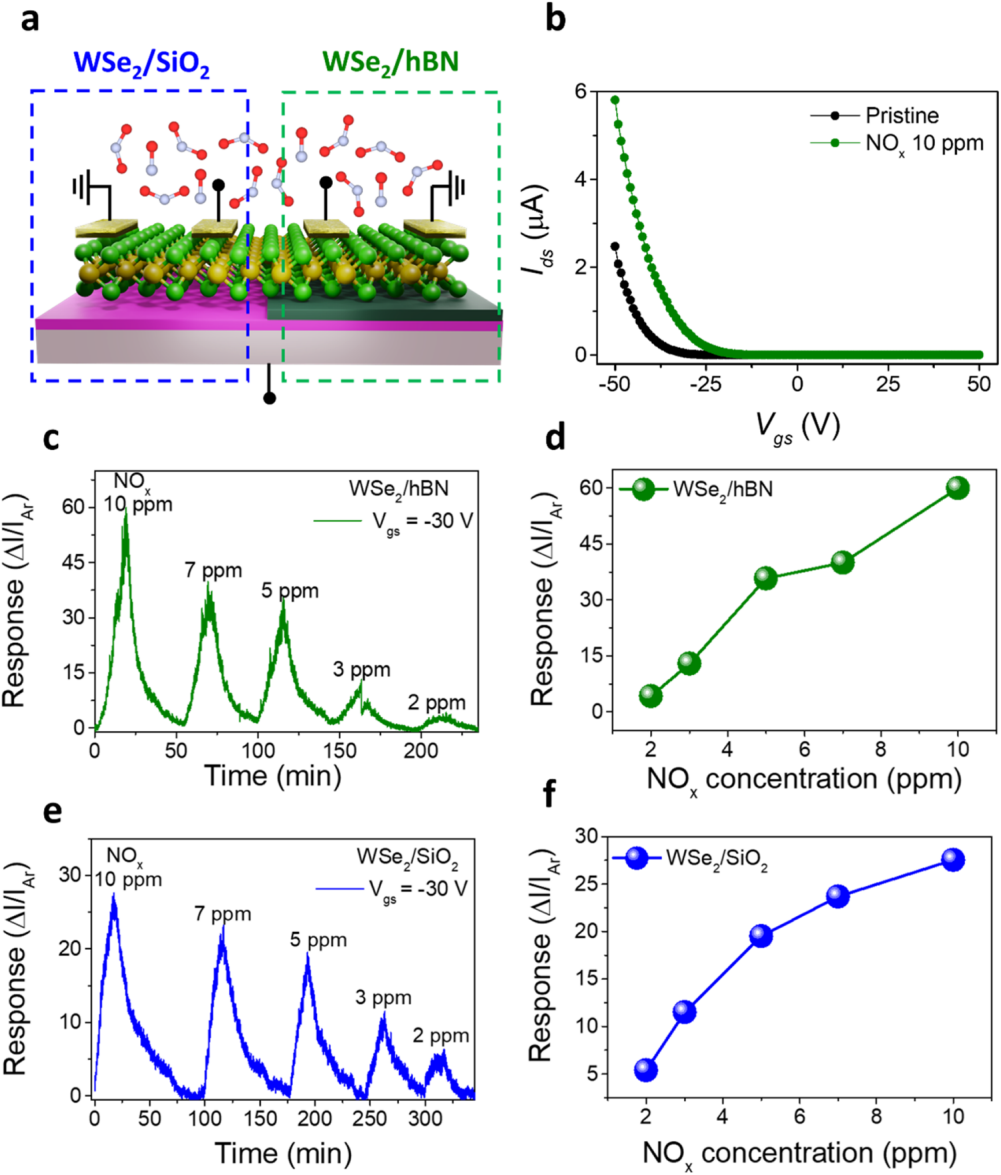
Electrical response of WSe_2_-based FETs to NO_*x*_ exposure. (a) Schematic representation of the FET structures, comparing WSe_2_/SiO_2_ and WSe_2_/hBN configurations under NO_*x*_ gas exposure. (b) Transfer characteristics of the WSe_2_/hBN FET before and after exposure to 10 ppm of NO_*x*_ gas. (c, e) NO_*x*_ response of WSe_2_/SiO_2_ and WSe_2_/hBN FETs for various concentrations ranging from 2 to 10 ppm. (d, f) Quantitative NO_*x*_ response as a function of gas concentration for both device configurations.

### 2.3 Surface Potential Dynamics of WSe_2_ FETs Under NO_*x*_ Exposure

Gas sensing in our FET gas sensor is attributed to a change in conductance of the channel material and a modification of the Schottky barrier at the contacts due to physisorption or chemisorption of the analyte gas molecules.^23,30^ Here we employed KPFM to measure the contact potential difference (*V*_CPD_) between the sensing film and KPFM tip by applying an external bias to the tip to compensate *V*_CPD_, allowing simultaneous acquisition of film topography and surface potential. The measured *V*_CPD_ can be directly used to map changes in the sample’s work function upon exposure to the NO_*x*_ gas. As discussed earlier, the FET gas sensing measurements demonstrated that WSe_2_/hBN devices exhibit enhanced sensitivity and relative selectivity toward NO_*x*_ gas compared to those of WSe_2_/SiO_2_ devices. Figure 3a depicts surface topography of the WSe_2_/hBN and WSe_2_/SiO_2_ devices, indicating atomically smooth surface with RMS roughness values of 0.6 and 0.84 nm, respectively. The In situ KPFM sensing measurements were performed on WSe_2_/hBN and WSe_2_/SiO_2_ FET devices at V_gs_ = 0 V and room temperature in a sequence of Ar gas, followed by NO_*x*_ (a mixture of 5 ppm of NO_2_ + 5 ppm of NO balanced in Ar) gas, and concludes with another round of Ar gas to mimic the complete exposure cycle of FET sensing. Figure 3b depict the surface potential map of the devices in the presence of Ar gas that indicate surface potential value of −90 mV in WSe_2_/hBN and −147 mV on WSe_2_/SiO_2_, indicating lower work function of WSe_2_/hBN, which can be due to the reduced Fermi level pinning on hBN substrate.^31^ After exposure to NO_*x*_ gas, an increase in the surface potential is observed in both devices, as shown in the surface potential map in Figure 3c and the corresponding line scans in Figure 3e. This increase can be attributed to the lowering of the Schottky barrier height (SBH) at the Au/WSe_2_ contact. It is well-known that the total change in device conductance, which translates into the sensing response, arises from both the channel and the contact region. The total device resistance is given in refs 30,32

1 $$R=R_{\mathrm{channel}}+R_{\mathrm{contact}}$$

2 $$R_{\mathrm{channel}}\propto \frac{1}{n}$$

3 $$R_{\mathrm{contact}}\propto {\frac{1}{n}e}^{\varphi \mathrm{SB}/\mathrm{kT}}$$

where *n* is the carrier concentration in the WSe_2_ channel, φSB is the Schottky barrier height (SBH), *k* is the Boltzmann constant, and *T* is the absolute temperature. Since both devices operate at *V*_gs_ = 0 V (i.e., the OFF state of the FET), the channel is depleted of charge carriers as evident from Figure 1(d),(e). Thus, the primary contribution to the change in conductance upon exposure to NO_*x*_ gas comes from the change in the SBH. The SBH at the Au/WSe_2_ interface can be qualitatively analyzed from the line scan of the SP map in Figure 3e.

**Figure 3:**
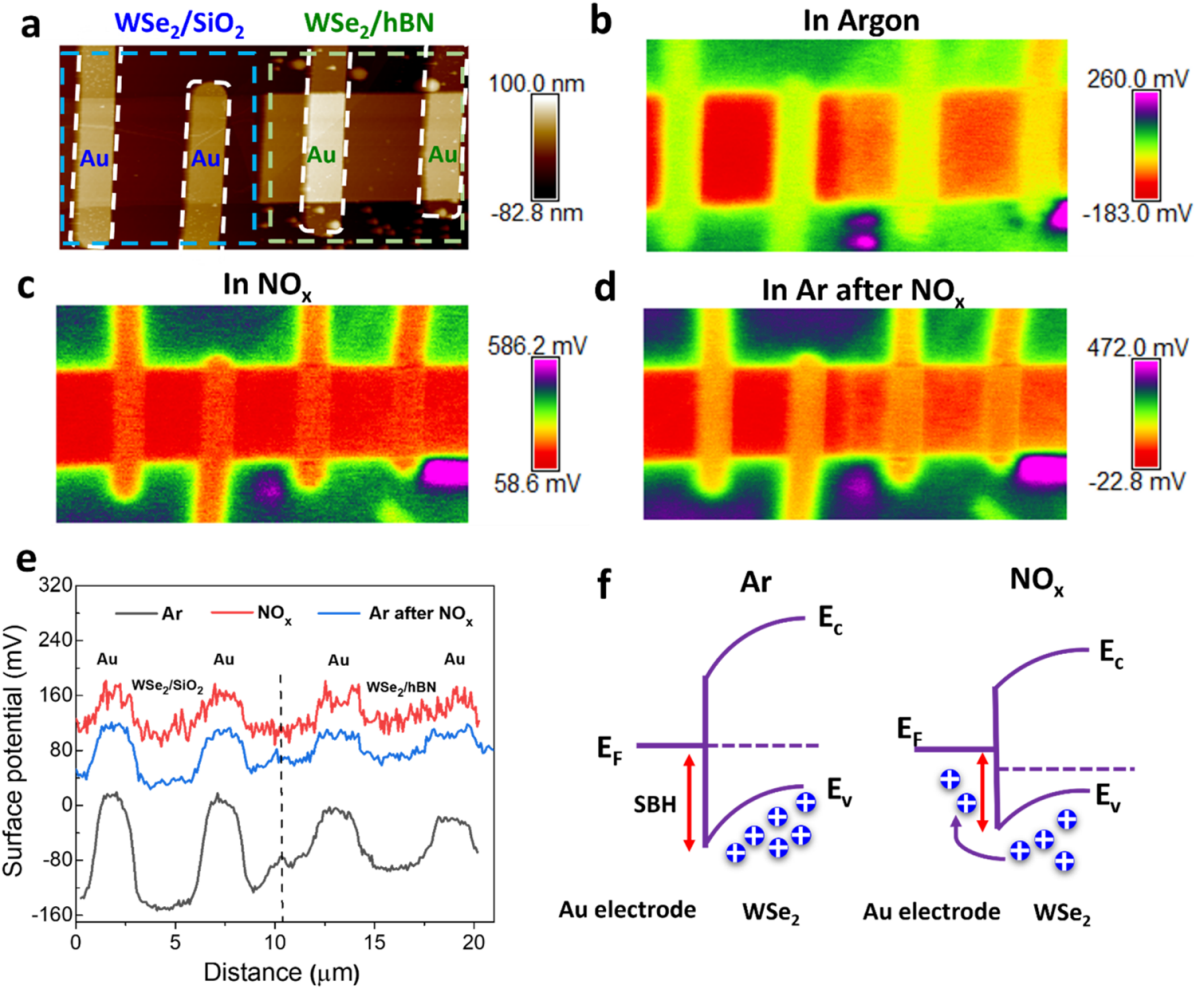
Surface potential and band diagram of WSe_2_ on SiO_2_ and the hBN substrate. (a) AFM topography image of the WSe_2_-based FET gas sensing device. The highlighted regions indicate the positions of WSe_2_ on SiO_2_/Si, WSe_2_ on hBN, and the Au electrodes. (b–d) Surface potential maps of the sensing device in the presence of Ar (b), NO_*x*_ gas (c), and again in Ar after removing NO_*x*_ gas (d). (e) The line profile of the surface potential across the device in the presence of Ar, NO_*x*_, and Ar after removing NO_*x*_. (f) Schematic illustrating the band bending in a WSe_2_ device due to NO_*x*_ at zero gate voltage.

A higher SBH is observed in the WSe_2_/SiO_2_ device contact compared to the WSe_2_/hBN device, which might be due to higher interface traps and weak dielectric screening in WSe_2_/SiO_2_. Upon exposure to NO_*x*_ gas, WSe_2_ undergoes p-type doping, causing a downward shift in the WSe_2_ Fermi level and a reduction in SBH, facilitating easier conduction across the contact (Figure 3f). Specifically, the SBH decreases from 164 to 50 mV in the WSe_2_/SiO_2_ device and from 87 to 44 mV in the WSe_2_/hBN device. The greater variation in the SB height of the WSe_2_/SiO_2_ device provides direct evidence that the contact region plays a dominant role in the overall sensing of the WSe_2_/SiO_2_ device. After NO_*x*_ is removed and Ar is reintroduced, the SP map starts to recover, as shown in Figure 3d,3e. This indicates a good but slow recovery, even at room temperature, suggesting that physisorption of gas molecules is the dominant interaction mechanism. However, complete recovery of the surface potential is observed after 24 h of Ar exposure, as shown in Figure S8. Additionally, annealing the device at 100 °C after NO_*x*_ exposure results in a similar recovery trend, with an overall decrease in SP. However, a key observation is the further reduction in SBH at 100 °C (Figure S8­(c)), which may be attributed to the increase in thermionic emission in the contact region at elevated temperatures.

### 2.4 UV-Induced Charge Modulation for Tunable NO_*x*_ Sensing in WSe_2_/hBN FETs

Furthermore, the effect of UV-induced charge modulation on the NO_*x*_ gas sensing properties of the WSe_2_/hBN devices was systematically studied. The transfer characteristics of the WSe_2_/hBN device were examined after UV writing at different gate voltages (+50 V, −20 V, and −50 V), revealing significant variations in carrier concentration and transport behavior (Figure 4a). This effect is attributed to the photoinduced electron doping mechanism in WSe_2_/hBN. Upon UV exposure, electrons trapped in defect states within hBN gain sufficient energy to transform into its conduction band. Under the influence of a negative gate voltage, these excited electrons are transferred to the conduction band of WSe_2_, resulting in n-type doping. Meanwhile, the positively charged defects left behind in hBN modify the local electrostatic environment, further influencing charge transport in WSe_2_.^31,33–35^ This redistribution of carriers plays a crucial role in modulating the gas sensing response as the carrier density in WSe_2_ determines the interaction strength with NO_*x*_ molecules, affecting charge transfer dynamics and overall sensing performance. For example, as observed in Figure 4a, devices written at +50 V exhibit increased hole conduction due to enhanced p-type doping, whereas those written at −50 V display electron doping characteristics. This modulation in charge carrier concentration directly correlates with the gas sensing response shown in Figure 4b, where devices written at +50 V demonstrate the highest NO_*x*_ sensitivity (∼100), while those written at −50 V exhibit no response for the same NO_*x*_ exposure (10 ppm). This behavior can be attributed to charge carrier redistribution and Schottky barrier modulation, which influence gas adsorption and charge transfer efficiency. In devices written at +50 V, hole accumulation in WSe_2_ enhances the interaction between the NO_*x*_ molecules and the channel. Since NO_*x*_ is an electron-withdrawing gas, it captures electrons from the semiconductor, further increasing the hole concentration and leading to a more pronounced sensing response. Additionally, the NO_*x*_ adsorption lowers the Schottky barrier for hole injection, further amplifying the sensing response. In contrast, in devices written at −50 V, where n-type doping dominates, the presence of excess electrons in WSe_2_ reduces the effectiveness of the NO_*x*_ adsorption. The charge transfer efficiency is limited as the electron-rich channel stabilizes charge redistribution, resulting in a diminished sensing response. Moreover, NO_*x*_ adsorption slightly increases the Schottky barrier for electron conduction, further restricting the sensing performance. Additionally, UV writing-induced shifts in the threshold voltage, I_ON_/I_OFF_ current ratio, and carrier mobility further influence the sensor’s performance, enabling an optically reconfigurable and highly adaptable gas sensing platform. It is noted that the recovery rate of the WSe_2_/hBN heterostructure slightly deteriorates after UV writing (Figure 4b), which may be attributed to the formation of UV-induced point defects in the WSe_2_ layer.^36^ These defects, such as chalcogen vacancies or trap states, can hinder the desorption of adsorbed NO_*x*_ molecules, thereby slowing the recovery kinetics.

**Figure 4:**
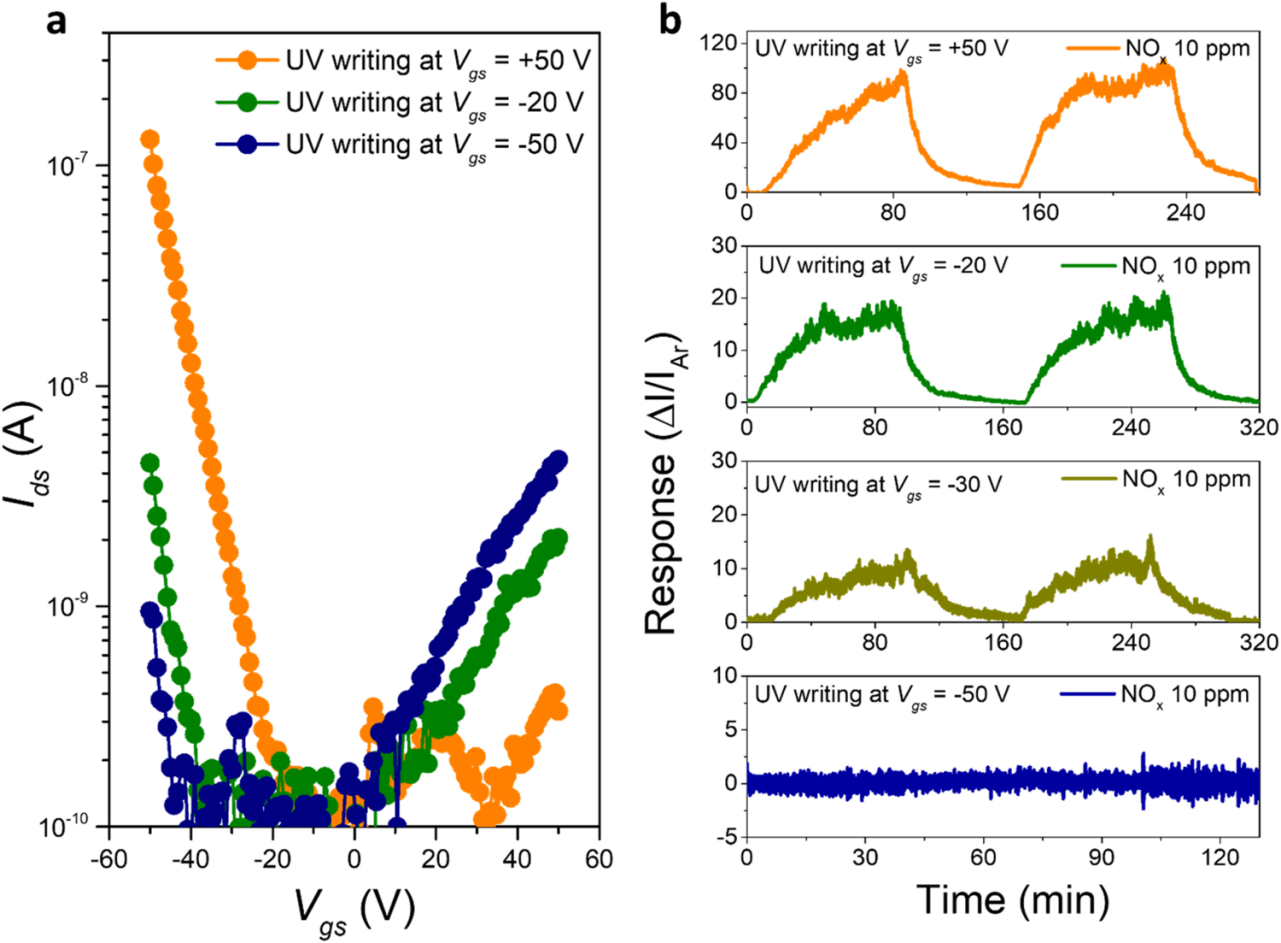
Optically tunable NO_*x*_ gas sensing with WSe_2_/hBN FETs. (a) Transfer characteristics of the WSe_2_/hBN FET at different writing gate voltages (*V*_is_ = +50 V, −20 V, and −50 V). (b) Corresponding NO_*x*_ gas sensing response recorded after each writing process. The NO_*x*_ sensing measurements were performed at an applied *V*_gs_ of −30 V and V_ds_ of 1 V under 10 ppm of NO_*x*_ exposure.

The stability of photoinduced doping in hBN-based heterostructures has been demonstrated in previous studies, showing long-term retention and reconfigurability under dark conditions,^31,33^ supporting the potential for stable operation of optically tunable gas sensors.

To benchmark our device performance, we compared it to recent reports on 2D material-based gas sensors. Table 1 summarizes the key metrics, including the device structure, working conditions, and sensing characteristics. Our WSe_2_/hBN-based optically tunable FET exhibits competitive advantages in terms of detection limit, tunability, response time, and room-temperature operation.

**Table 1:** Comparison of Gas Sensing Performance of 2D Material-Based Sensors with the Present WSe_2_/hBN FET Device

2D material	morphology	device type	gas concentration (ppm)	working temperature (°C)	response (%)	LoD (ppm)	gas detected	response time	refs
WSe_2_	nanoflower	chemiresistive	0.8 ppm	100	20.5	0.1	NO_2_	196 s	^37^
WSe_2_	monolayer	chemiresistive	2	RT	18.8		NO_2_	24 min	^38^
WSe_2_	trilayer	chemiresistive	500	RT	4140		NO_2_	15 min	^39^
WSe_2_	nanoflower	chemiresistive	2	120	45	0.1	NO_2_		^40^
MoS_2_	four layers	FET	5	RT	59.4	5	NO_2_	24s	^41^
WSe_2_	thin film	chemiresistive	20	250	88.89	0.1	NO	109s	^42^
WSe_2_	liquid exfoliated nanosheet	chemiresistive (optically activated)	10	RT	436	0.08	NO_2_		^43^
WSe_2_	bulk flake	FET	1	RT	350	1	NO_2_		^44^
MoS_2_	bilayer	FET	200	RT	100	100	NO_2_		^45^
WSe_2_ (hBN substrate)	few layers (8 layers)	FET (optically tunable)	10	RT	6000 (in %), tunable (10,000– 0)	<2	NO_*x*_ (NO_2_+NO)	12 min	this work

The ability to optically modulate the charge carrier concentration in WSe_2_/hBN FETs offers a promising route toward the development of smart gas sensors with dynamic selectivity. By performing UV writing at controlled gate biases, the baseline doping level of the device can be precisely tuned, enabling the sensor to respond differently to various gas species depending on their electron-donating or -withdrawing nature. This tunability allows the sensor to distinguish between different types of gases (e.g., NO_*x*_ vs NH_3_ or VOCs) based on their interaction profiles with either p-type or n-type channels. Integrating this capability into a reconfigurable platform could pave the way for next-generation intelligent sensing systems that can adaptively identify gas types through a combination of electrical and optical inputs, offering enhanced selectivity, sensitivity, and versatility in real-time environmental monitoring.

## 3 Conclusions

In summary, we have demonstrated an optically programmable WSe_2_/hBN heterostructure transistor-based gas sensor with enhanced sensitivity, and response speed for NO_*x*_ detection. The hBN interfacial layer played a crucial role in enhancing charge transport by minimizing charge trapping and interface defects, leading to a superior sensing performance compared with WSe_2_/SiO_2_ devices. Our in situ KPFM measurements provided direct evidence that NO_*x*_ adsorption modulates the Schottky barrier height (SBH), influencing higher charge injection and transport dynamics in the WSe_2_/SiO_2_ device, and it can effectively be employed to isolate the contribution of the channel and contact region. Additionally, we introduced UV-induced charge modulation as a method to dynamically tune the sensor response, offering a reversible and adaptive approach to optimizing gas detection. This work paves the way for next-generation smart sensing technologies with potential applications in environmental monitoring, industrial safety, and smart sensor networks.

## 4 Experimental Section

### 4.1. Device Fabrication

A few-layer WSe_2_ flake was mechanically exfoliated and transferred onto a around 60 nm-thick hBN flake and a 300 nm SiO_2_ substrate using a dry transfer method (Figure S1), ensuring that the flake was positioned across both substrates. The WSe_2_ channel was defined through electron-beam lithography (EBL) and reactive ion etching (RIE). Subsequently, EBL and electron-beam evaporation were employed to fabricate the electrodes, followed by a lift-off process using titanium/gold (5/110 nm) contacts.

### 4.2. Electrical Characterization

The electrical measurements of the devices were conducted by using a two-point measurement setup with a Keithley 2440 source meter for current–voltage measurements. The gate voltage was controlled using a Keithley 2450 instrument, and all data acquisition was automated through a customized LabVIEW program. The field-effect carrier mobility (μ) was calculated by using eq 4.

4 $$\mu =\left[{dI}_{\mathrm{ds}}/{dV}_{g}\right]\times \left[L/\left({\mathrm{WC}}_{t}V_{\mathrm{ds}}\right)\right]$$

where d*I*_ds_/d*V*_g_ represents the transconductance derived from the transfer characteristics, *L* and *W* correspond to the channel length and width, *C*_*t*_ denotes the total capacitance per unit area, and *V*_ds_ is the applied drain-source voltage.

### 4.3. Gas Sensing Measurements

Gas sensing measurements were carried out in a custom-designed test chamber equipped with mass flow controllers to ensure controlled gas flow. The sensing chamber was flushed with argon (Ar) to establish a stable baseline prior to the target gas exposure. A 50/50 mixture of NO_2_ and NO was used as the NO_*x*_ source in this work. Prediluted gas cylinders with concentrations of 5 and 5 ppm (balanced in argon) were obtained from Linde Gas AS (Norway). To achieve the required concentrations for the experiments, the NO_*x*_ gases were further diluted by mixing with argon before being introduced into the gas chamber. The total gas flow rate was maintained between 40 and 80 mL/min.

The sensing performance was evaluated by monitoring the drain current (*I*_ds_) of the devices under alternating exposure to argon (Ar) and NO_*x*_ gases. The devices were biased with a drain voltage *V*_ds_ = 1 V and a gate voltage *V*_gs_ = −30 V.

The sensing response was calculated using the formula

5 $$\mathrm{response}=\left(I_{NO_{x}}-I_{\mathrm{Ar}}\right)/I_{\mathrm{Ar}}$$

where *I*_No*_x_*_ is the current under *NO*_*x*_ exposure and *I*_Ar_ is the baseline current in Ar.

The response time (τ_res_) is defined as the time required to reach 90% of the total current change upon NO_*x*_ exposure, while the recovery time (τ_rec_) is defined as the time to return to 10% of the total change after gas removal.

### 4.4. In situ Kelvin Probe Force Microscopy

In situ KPFM measurements were performed at room temperature using a Bruker Multimode 8 atomic force microscope with a gas cell attachment, as illustrated in Figure S7. The system was equipped with a conductive Pt–Ir-coated cantilever for electrical surface potential measurements. The cantilever used had a tip radius of 35 nm, a spring constant of ∼0.4 N/m, and a resonance frequency of ∼70 kHz. The KPFM scans were carried out by using a two-pass amplitude modulation (AM) mode. In the first pass, the surface topography of the sample was recorded in an intermittent contact (tapping) mode, allowing for accurate profiling of nanoscale features. Following this, the second pass was executed at a constant lift height of 100 nm above the sample surface. During this lift mode, the mechanical oscillation of the cantilever was halted to isolate electrostatic interactions from the topographic contributions. To probe the local contact potential difference (CPD) between the tip and the sample surface, an AC voltage and a frequency close to the cantilever’s resonance were applied between the tip and the sample. If a potential difference existed, then an oscillating electrostatic force was induced, causing the cantilever to vibrate. A feedback loop in the system then applied a compensating DC bias voltage to nullify the oscillation amplitude. This applied DC voltage effectively corresponded to the local surface potential at each scanned point. The scans were acquired at a rate of 1 Hz with a spatial resolution of 256 pixels per line, ensuring sufficient detail. The probe tip was calibrated prior to each set of measurements using a highly oriented pyrolytic graphite (HOPG) reference sample. Calibration was performed under environmental and instrumental conditions identical to those used during actual in situ KPFM measurements.

### 4.5. TEM Characterization

TEM samples were prepared by using a Helios G4 UX dual-beam focused ion beam (FIB) system. A 3 μm-thick carbon layer was initially deposited over the region of interest before the TEM lamella. The first layer of carbon was applied using electron-beam-assisted deposition to minimize potential Ga^+^ ion damage in the target area. For ion beam thinning, a 30 kV acceleration voltage was used for initial coarse thinning, followed by final thinning at 5 and 2 kV on both sides of the lamella to reduce surface damage. TEM imaging was carried out at 200 kV using a double Cs aberration-corrected cold field-emission JEOL ARM 200FC microscope.

## Supplementary Material



## Notes

Heterostructure fabrication process, STEM image of WSe_2_ on SiO_2_ substrate, output characteristic curves of WSe_2_/SiO_2_ and WSe_2_/hBN FETs, transfer characteristics before and after exposure to 10 ppm of NO*_x_* gas, time-resolved electrical response and recovery of devices, schematic diagram of in situ KPFM setup, surface potential maps after NO*_x_* removal and postheating at 100 °C (PDF)
