# Supplementary material for: Optically Programmable Smart WSe2/hBN Heterostructure Gas Sensors
Source: ACS Appl Mater Interfaces. 2025 Aug 12;17(36):50977–85. doi: 10.1021/acsami.5c09390 (PMC12442008; doi:10.1021/acsami.5c09390)
Supplement: Supplementary file 1 [file am5c09390_si_001.pdf]

Supporting Information

Optically Programmable Smart WSe_2/hBN Heterostructure Gas Sensors

*Ayaz Ali[†‖], Prashant Bisht[‼⊥], Matthias Schrade[‡], Wen Xing[‡], Per Erik Vullum[#], Takashi Taniguchi[¶], Kenji Watanabe[§], Bodh Raj Mehta[⊥¤] and Branson Delano Belle[‡$]**

[†]Department of Cybernetics, Nanotechnology and Data Processing, Faculty of Automation Control, Electronics and Computer Science, Silesian University of Technology, Akademicka 16, 44-100 Gliwice, Poland

[‖]Department of Smart Sensor Systems, SINTEF DIGITAL, Forskningsveien 1, Oslo 0373, Norway

[‼]School of Advanced Materials Science and Engineering, Sungkyunkwan University, Suwon 16419, Republic of Korea

[‡]Department of Sustainable Energy Technology, SINTEF, Forskningsveien 1, Oslo 0373, Norway

[#]Department of Materials and Nanotechnology, SINTEF, Høgskoleringen 5, Trondheim 7034, Norway

[¶]Research Center for Materials Nanoarchitectonics, National Institute for Materials Science, 1-1 Namiki, Tsukuba 305-0044, Japan

[§]Research Center for Electronic and Optical Materials, National Institute for Materials Science, 1-1 Namiki, Tsukuba 305-0044, Japan

[⊥]Department of Physics, Indian Institute of Technology Delhi, New Delhi 110016, India

[¤]Directorate of Research, Innovation and Development, Jaypee Institute of Information Technology. Noida (U.P.), 201309, India

[$]Centre for Oceanography and the Blue Economy, University of the West Indies, Five Islands, Antigua and Barbuda

**Address correspondence to:* branson.belle@uwi.edu

S1. Heterostructure fabrication process

The fabrication process for the WSe_2/hBN 2D heterostructure is schematically illustrated in Figure S1. First, a WSe_2 flake was mechanically exfoliated onto a PMMA/PVA-coated silicon substrate (step 1), as shown in Figure S1a. The WSe_2/PMMA stack was then separated from substrate 1 by dissolving the sacrificial PVA layer, which acted as an intermediate between PMMA and the silicon surface. Meanwhile, an hBN flake was mechanically exfoliated onto a Si/SiO_2 substrate (step 2), as depicted in Figure S1b. Finally, the WSe_2/PMMA stack was flipped, aligned, and precisely transferred onto the hBN flake under an optical microscope (step 3 and Figure S1c). The PMMA support layer was then removed by dissolving it in acetone, completing the heterostructure fabrication.

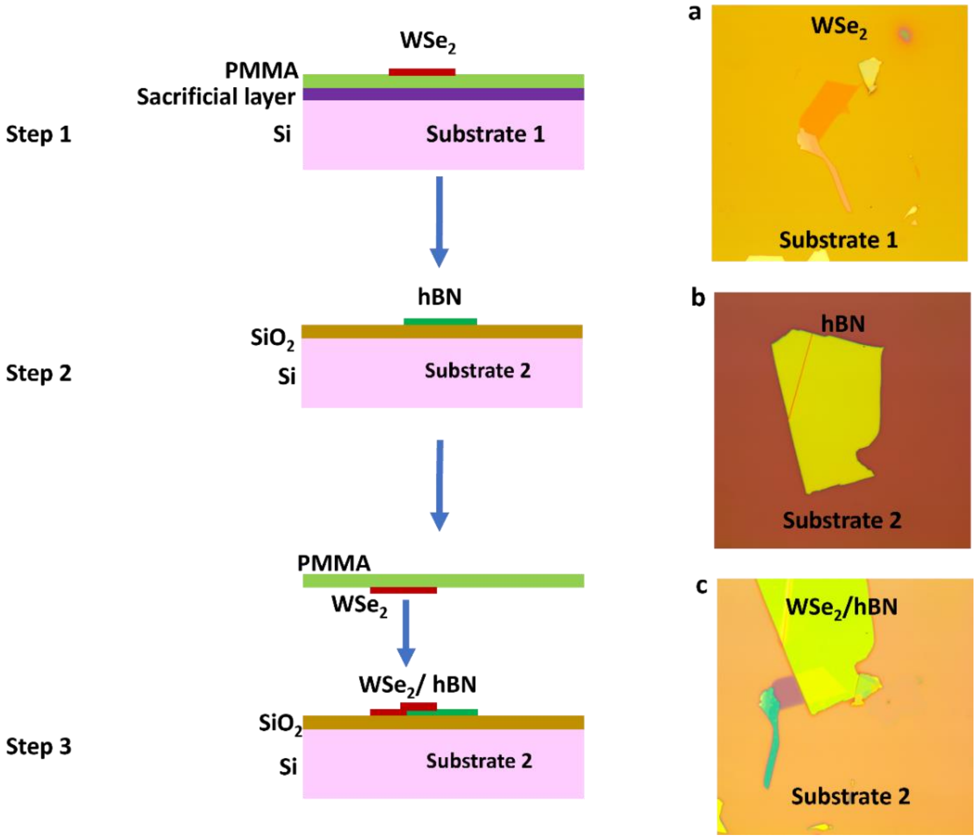

Figure S1. WSe_2/hBN Heterostructure fabrication process. (a) optical image of WSe_2 flake on Si substrate coated with a sacrificial layer and PMMA, (b) optical image of an hBN flake on Si/SiO_2 substrate, (c) optical image of the WSe_2 flake transferred on the hBN flake.

S2. Bright field TEM image of WSe_2/SiO_2

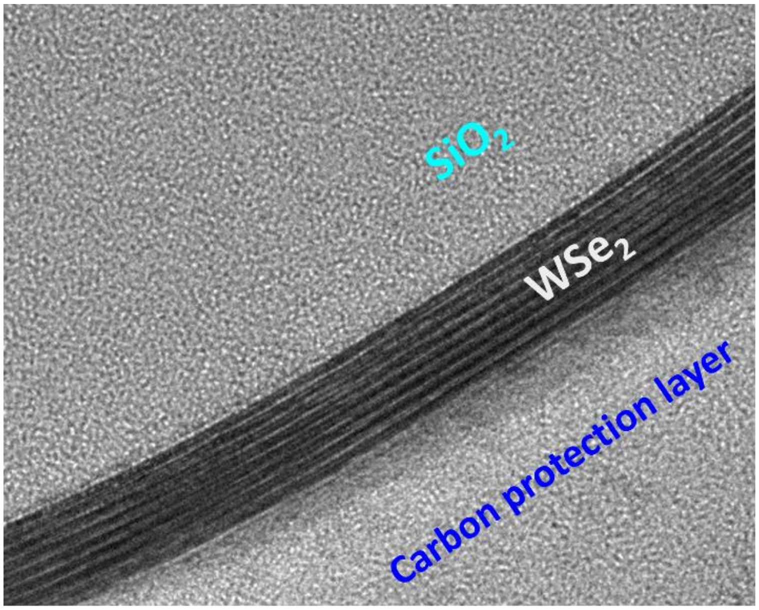

Figure S2. Bright field TEM image of WSe_2/SiO_2 region of as fabricated device

S3. Output characteristics of WSe_2/SiO_2 and WSe_2/hBN FETs

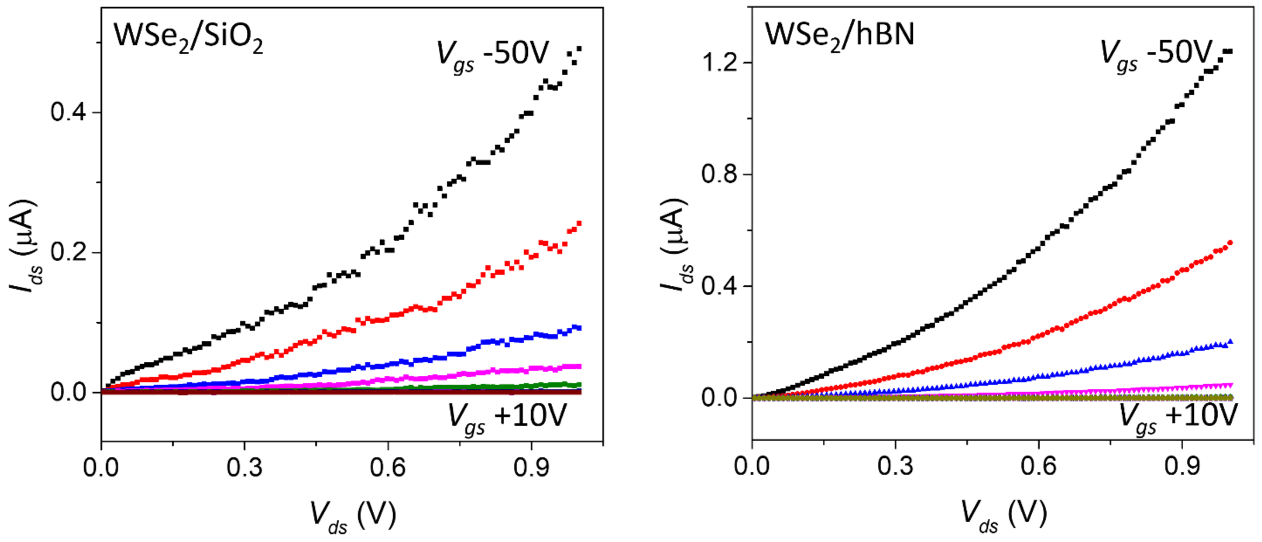

Figure S3. Output characteristics of as fabricated pristine WSe_2/SiO_2 and WSe_2/hBN FETs under different gate bias (V_{gs} = +10 V to -50 V).

S4: Logarithmic-scale transfer characteristics of the WSe_2/hBN pristine device and after exposure to 10 ppm NO_x gas.

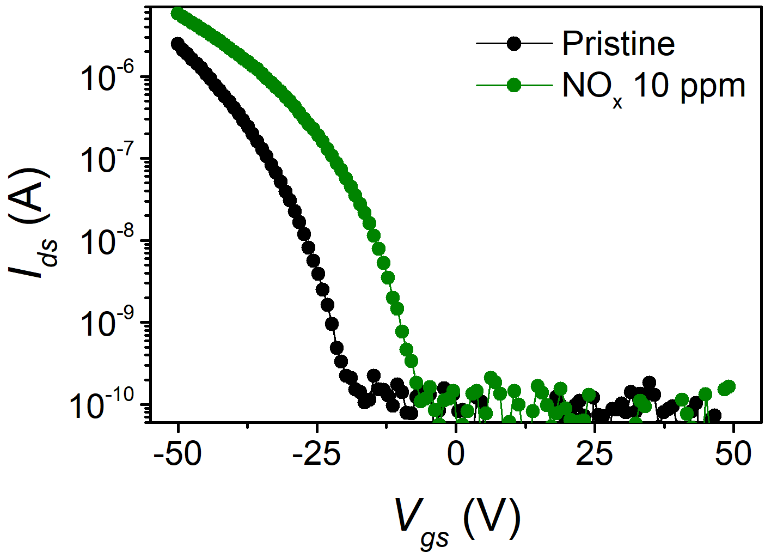

Figure S4. Logarithmic-scale transfer characteristics of the WSe_2/hBN pristine device (black curve) and after exposure to 10 ppm NO_x gas (green curve), measured at V_{ds} = 1V. The plot clearly reveals a positive shift in the threshold voltage (V_{th}), indicating effective charge transfer and doping effects induced by NO_x adsorption.

S5. Transfer characteristics of the WSe_2/SiO_2 FET before and after exposure to 10 ppm NO_x gas

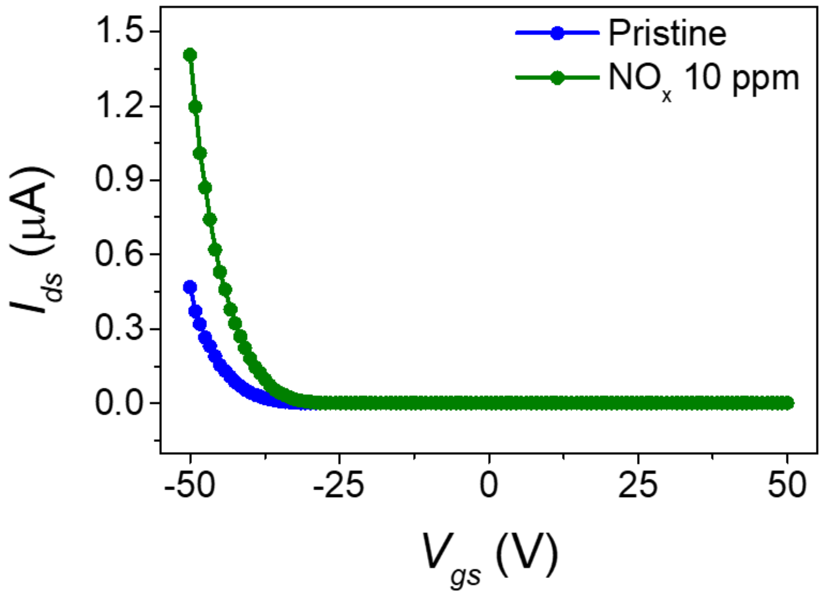

Figure S5. Transfer characteristics of the WSe_2/SiO_2 FET before and after exposure to 10 ppm NO_x gas.

S6. Time-resolved electrical response and recovery of WSe_2/hBN and WSe_2/SiO_2 devices

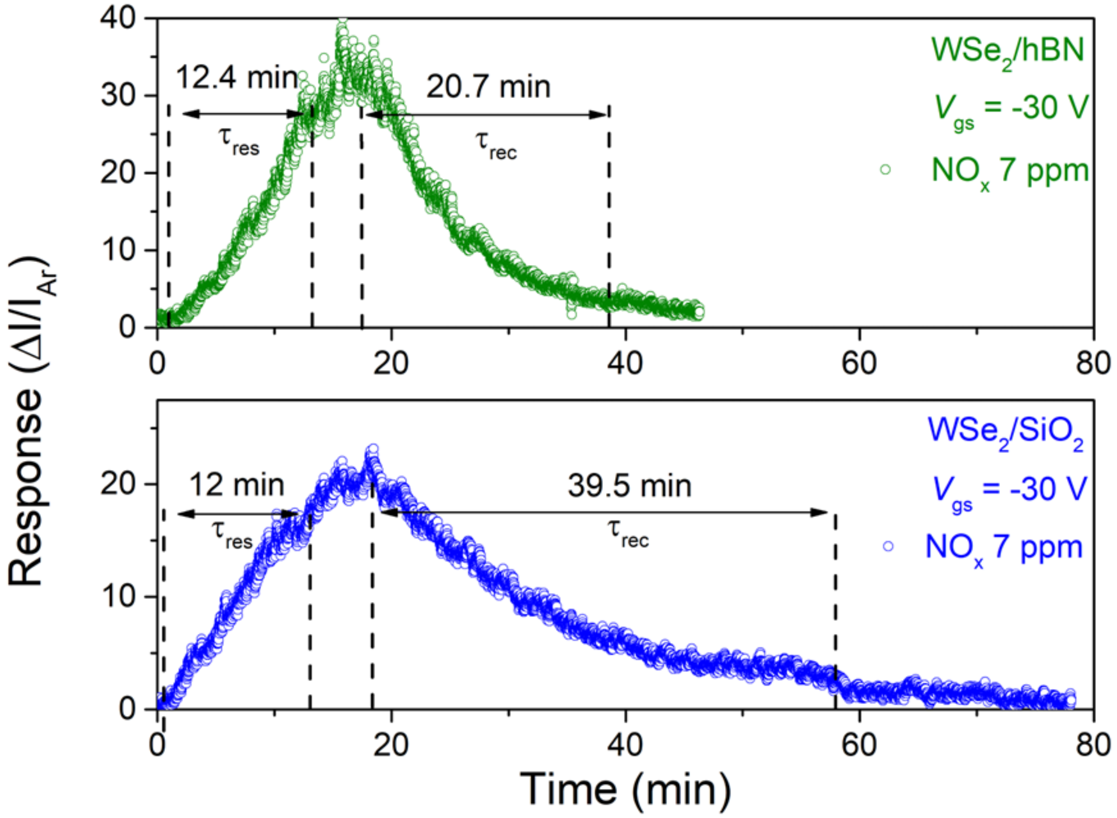

Figure S6. Time-resolved electrical response and recovery of WSe_2/hBN and WSe_2/SiO_2 devices upon exposure to 7 ppm NO_x at V_{gs}=−30 V

S7. In-situ Kelvin Probe Force Microscopy setup

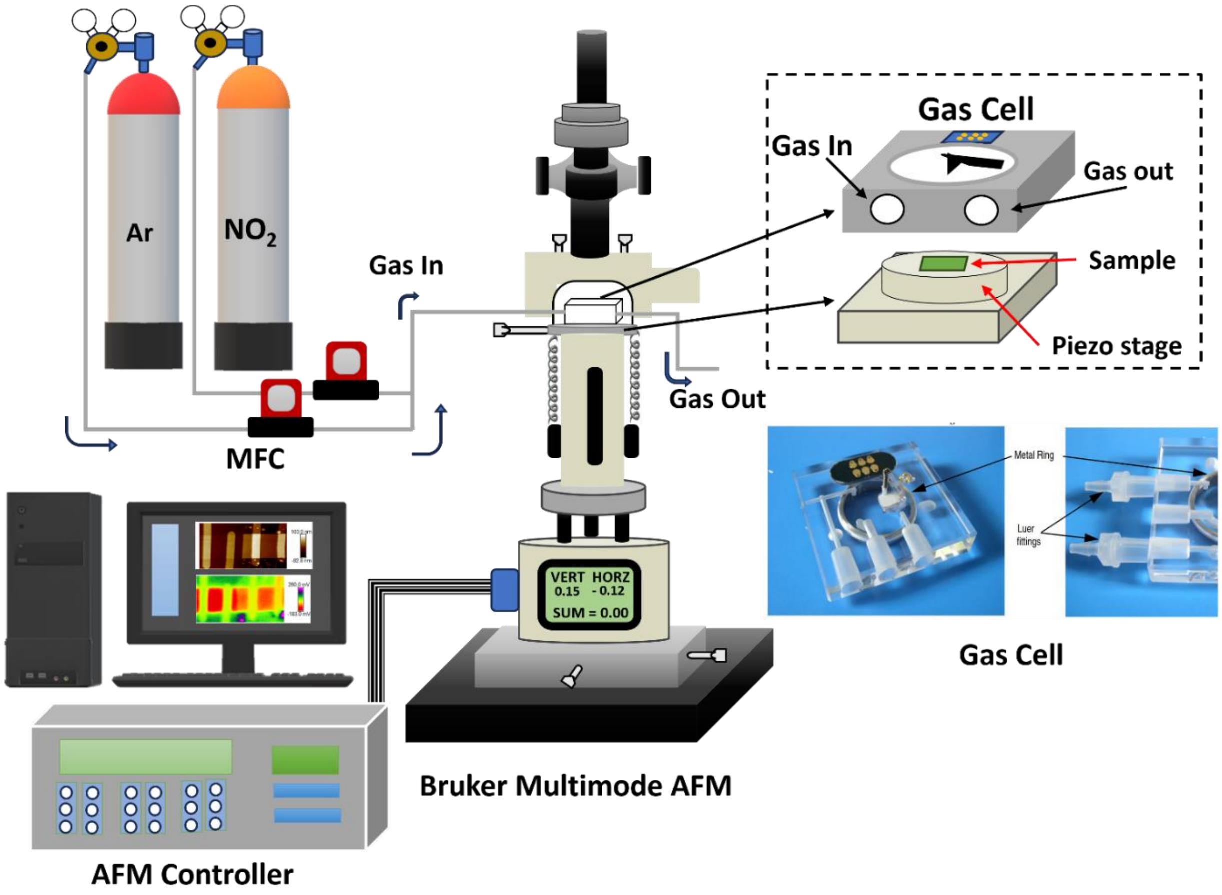

Figure S7: Schematic diagram of the In-situ KPFM setup

S8. Surface potential maps of the sensing device in different conditions

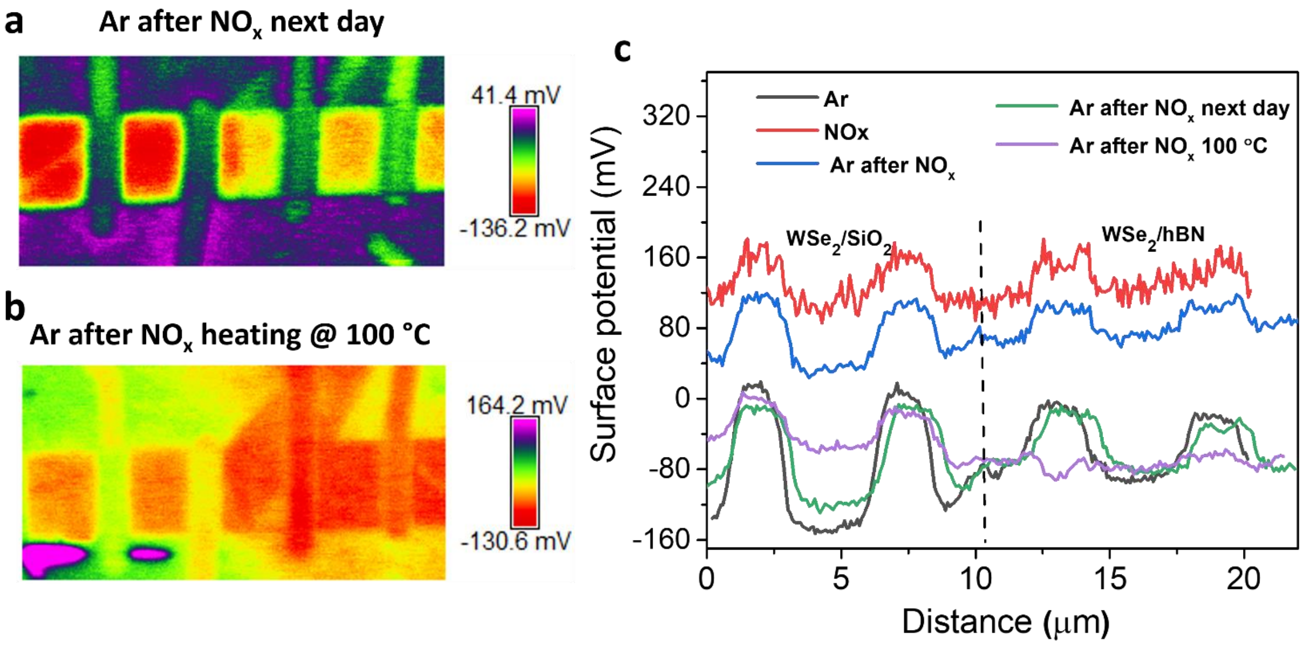

Figure S8: Surface potential maps of the sensing device: (a) 24 hours after the removal of NO_x gas and (b) after heating the device at 100 °C following NO_x gas removal. (c) Line profile of the surface potential across the device under different conditions: in the presence of Ar, NO_x, Ar after NO_x removal, 24 hours after NO_x removal, and after annealing at 100 °C post NO_x removal.
